# An Accessible Intersectional Transgenic Single-Vector CRISPR/Cas9 Platform for Precise Gene Editing and Functional Analysis

**DOI:** 10.1523/ENEURO.0264-24.2024

**Published:** 2024-08-02

**Authors:** Carlee A. Toddes

**Affiliations:** Department of Biological Structure, University of Washington, Seattle, Washington 98195

In 1987, while investigating the iap gene in *Escherichia coli*, Nakata and colleagues identified “five highly homologous sequences of 29 nucleotides were arranged as direct repeats with 32 nucleotides as spacing.” Perplexed by this discovery, the authors stated, “so far, no sequence homologous to these has been found elsewhere in prokaryotes, and the biological significance of these sequences is not known.” These few sentences launched a decades-long investigation into the biological mystery of clustered regularly interspaced short palindromic repeats, or CRISPR (Ishino et al., 1987).

Since its first identification, CRISPR—an adaptive immune response in prokaryotic organisms to recognize and combat infectious DNA—has been developed into a tool for targeted gene editing in a variety of cells and organisms (Jansen et al., 2002). The utility of CRISPR lies in the easy targeting of virtually any genomic location by a short RNA guide. In its simplest form, the two components that must be expressed in cells to perform genome editing are the Cas9 nuclease and a guide RNA (gRNA; Hille and Charpentier, 2016). Due to this simplicity, high efficiency and multiplex genome editing capabilities, CRISPR-Cas has become a highly relied upon editing tool.

As the development of effective, reliable, and customizable in vivo gene editing technologies advanced, so too did genetically encoded tools for visualizing and manipulating cellular function in vivo. Optogenetics and several other transgene technologies—fiber photometry, chemogenetics, and one-photon imaging—emerged in the early 2000s, enabling optical recording or manipulation of specified cell types. While targeted in vivo genetic editing reveals the impact of specific genes on nervous system functions and diseases, tools like optogenetics allow investigation of the functional roles of cells and circuits in behavior. The next logical step in the progression of these technologies was to combine gene editing with transgene expression, facilitating groundbreaking insights into how genes regulate cellular and circuit activity.

Currently, however, platforms that combine CRISPR-Cas with cell-specific transgene expression for neuronal visualization or manipulation are inadequate. The large Cas enzyme and limited packaging space of AAVs prevent reliable copackaging of transgenes with Cas, necessitating a two-vector transduction system (Swiech et al., 2015). However, coinjection of AAVs is often inefficient. The recent *eNeuro* article by Moffa et al. (2024) has addressed this issue by developing a platform that combines Cre-dependent Cas9 mouse lines with Cre-driver lines, enabling cell type-specific Cas9 expression (Platt et al., 2014). Now, a single viral vector containing a targeted gRNA and a transgene of choice can be used to achieve an edited neuronal population that fully overlaps with transgene expression. This platform provides a robust approach to test novel genes for effects on synaptic transmission, circuit activity, morphology, and behavior.

To test the utility of a single-vector approach for transgene expression and CRISPR-Cas9 editing, Moffa et al. (2024) carefully designed three discrete constructs where either ChRonos, GCaMp8f, or mCherry were inserted into modified viral vectors along with gRNA against Vgat, GRIN1, or Dicer genes. These constructs were then injected into hybrid Vgat-Cre:IsI-Cas9, TH-Cre:ISI-Cas9, or PV-Cre:ISI-Cas9 mice, restricting Cas9 expression to GABAergic, dopaminergic, or parvalbumin expressing neurons, respectively. These experiments permitted targeted manipulation or visualization of selected neuronal populations and anatomical tracing of peripheral proprioceptive neurons.

The authors first target the GABA transporter (*Vgat*) in the nucleus accumbens (NAc) for genetic editing. Simultaneously, GABAergic neurons were targeted for cre-dependent expression of the channelrhodopsin ChRonos. Immunohistochemistry (IHC) of experimental animals revealed significantly fewer VGAT puncta compared with control mice, confirming anatomical alterations caused by gene editing. Additionally, electrophysiological recordings revealed significantly reduced amplitudes of optically evoked IPSCs in experimental animals compared with controls. These data demonstrate the exciting utility of a single-vector approach for gene editing, neuron visualization, and functional investigation using optogenetics.

Next, the authors validated this single-vector approach for selective recording of the neuronal activity of edited neurons. NMDA receptors in dopaminergic neurons of the ventral tegmental area (VTA) were targeted with a gRNA against the Grin1 subunit of NMDA while Cre-dependent GCaMP8f permitted fiber photometry recordings of edited neurons. Interestingly, GCaMP8f Ca^2+^ transients in the VTA of awake, behaving experimental animals were nearly absent compared with control animals demonstrating effective knockdown of Grin1. These functional alterations were confirmed via ex vivo electrophysiological recordings of edited neurons. These results demonstrate that the CRISPR-Cas9 platform can be effectively used in combination with fiber photometry to selectively monitor activity in genetically edited neurons.

Lastly, the authors sought to demonstrate the efficacy of their single-vector platform in the peripheral nervous system, which is typically excluded from gene editing experiments. To target the gene Dicer in parvalbumin-positive peripheral neurons, a Cre-dependent mCherry transgene and gRNA targeted to Dicer were packaged into a PNS-selective PHP.S serotyped AAV. This construct was then retro-orbitally injected into PV-Cre/ISI-Cas9 mice. This manipulation showed high infection efficiency with no off-target expression and a significant reduction in the fluorescence density and area innervated by mCherry+ spinal cord projections of PV+ neurons in edited compared with control mice in the spinal cord.

The single-vector platform devised by Moffa et al. (2024) offers broad utility and efficacy for CRISPR-Cas9 gene editing and expression of genetically encoded tools in both the central and peripheral nervous systems. Combined with optogenetic techniques, it enables thorough investigation of neurotransmitter release, learning, and synaptic plasticity. Moreover, when paired with in vivo genetically encoded biosensor imaging, it facilitates understanding of specific genetic influences on neural circuit dynamics. Integration with fluorescent proteins supports studies on genetic drivers of neuronal morphology, connectivity, and survival. Using Cre-dependent Cas9 mouse lines instead of packaged Cas9 allows for additional gRNAs, improves targeting of a single gene, and enables targeting of multiple genes simultaneously.

While promising, this platform has technical limitations including its primary usefulness being limited to mice, potential for leaky Cre expression during development, and restriction to genes with one or few transcript variants. Ongoing developments in CRISPR technology aim to address these limitations, such as incorporating U6-gRNAs to mitigate leaky Cre expression and multiple gRNAs in a single vector to target all gene variants. Overall, the single-vector CRISPR/Cas9 approach offers an accessible, flexible, and precise mechanism for gene function interrogation in specific cell types and circuits across the nervous system.

## Author’s Note

All CRISPR single viral vectors used in Moffa et al. (2024) are available on Addgene.
